# A novel neural network-based framework to estimate oil and gas pipelines life with missing input parameters

**DOI:** 10.1038/s41598-024-54964-3

**Published:** 2024-02-24

**Authors:** Nagoor Basha Shaik, Kittiphong Jongkittinarukorn, Watit Benjapolakul, Kishore Bingi

**Affiliations:** 1 Department of Mining and Petroleum Engineering, Faculty of Engineering, Chulalongkorn University, Bangkok, 10330 Thailand; 2 Center of Excellence in Artificial Intelligence, Machine Learning and Smart Grid Technology, Department of Electrical Engineering, Faculty of Engineering, Chulalongkorn University, Bangkok, 10330 Thailand; 3 Department of Electrical and Electronic Engineering, Universiti Teknologi PETRONAS, 32610 Seri Iskandar, Perak Malaysia

**Keywords:** Artificial neural networks, Correlation analysis, Missing data, Oil and gas, Pipelines, Reliability, Mechanical engineering, Civil engineering

## Abstract

Dry gas pipelines can encounter various operational, technical, and environmental issues, such as corrosion, leaks, spills, restrictions, and cyber threats. To address these difficulties, proactive maintenance and management and a new technological strategy are needed to increase safety, reliability, and efficiency. A novel neural network model for forecasting the life of a dry gas pipeline system and detecting the metal loss dimension class that is exposed to a harsh environment is presented in this study to handle the missing data. The proposed strategy blends the strength of deep learning techniques with industry-specific expertise. The main advantage of this study is to predict the pipeline life with a significant advantage of predicting the dimension classification of metal loss simultaneously employing a Bayesian regularization-based neural network framework when there are missing inputs in the datasets. The proposed intelligent model, trained on four pipeline datasets of a dry gas pipeline system, can predict the health condition of pipelines with high accuracy, even if there are missing parameters in the dataset. The proposed model using neural network technology generated satisfactory results in terms of numerical performance, with MSE and R^2^ values closer to 0 and 1, respectively. A few cases with missing input data are carried out, and the missing data is forecasted for each case. Then, a model is developed to predict the life condition of pipelines with the predicted missing input variables. The findings reveal that the model has the potential for real-world applications in the oil and gas sector for estimating the health condition of pipelines, even if there are missing input parameters. Additionally, multi-model comparative analysis and sensitivity analysis are incorporated, offering an extensive comprehension of multi-model prediction abilities and beneficial insights into the impact of various input variables on model outputs, thereby improving the interpretability and reliability of our results. The proposed framework could help business plans by lowering the chance of severe accidents and environmental harm with better safety and reliability.

## Introduction

A dry gas pipeline system transports natural gas from the oil field to processing and warehousing facilities or individual customers^1^; unlike wet gas pipelines, which transport liquids like butane and propane, dry gas pipelines solely transport methane, constituting the principal component of natural gas^2^. The pipeline system consists of pipes, processing plants, and regulation stations to manage the flow and pressure of gas^3^. Dry gas pipelines transmit enormous amounts of natural gas across great distances, offering a consistent and efficient means of meeting the expanding reliance on fossil fuels as an energy source. Dry gas pipelines have faced several operational, technical, and environmental issues for many years^4^. Some of the most typical issues are Corrosion: Pipelines can corrode and deteriorate over time, limiting their lifespan and creating safety issues^5,6^. Spills and leaks: The spills and leaks may be hazardous to the environment and cause costly cleaning and repairs^7^. Regulation: The pipeline sector is subject to various rules and standards, which can be complicated and continuously changing, making compliance challenging for operators^8^. Cybersecurity concerns: With the increased use of technology in pipeline systems, they have become increasingly exposed to cyber assaults^9^. A good strategy for better maintenance, management, and creative techniques is required to address these difficulties and increase safety, durability, and efficiency.

Several studies have been carried out to forecast the lifetime of oil and gas pipelines. Material Degradation Analysis (MDA), Finite Element Analysis (FEA), Condition-Based Monitoring (CBM), Probabilistic Risk Assessment (PRA), Machine Learning (ML) and Artificial Intelligence (AI), and specific data-driven methodologies are some of the techniques commonly employed in these investigations^10^. Their investigations marked out the research and application of oil and gas pipeline failure prediction and revealed their deficiencies in proposing future research areas. Furthermore, this study gave insights into the effort to select appropriate failure prediction methods for incorporation into present evaluation measures. MDA investigates in depth the impacts of corrosion, erosion, and other elements that might cause material deterioration and impair the service life of the pipeline. MDA has been used in specific research^11^. This study conducted a metallurgical investigation, including compositional, microstructural, and hardness tests. Experimental findings were compared to field data to aid in establishing the deterioration mechanism. The study concluded that the concentration and kind of corrosion inhibitor have a vital impact on its performance, showing the significance of metal loss type on pipeline life degradation. Few researchers employed the finite element analysis (FEA) approach to estimate the behaviour of a pipeline over load variations and operating situations^12^. This study investigates the various types of corrosion faults and the influence of dimensional loss on estimating pipeline failure pressure using FEA. They concluded that the failure pressure could be predicted more precisely by considering both corrosion defects and variances in material properties, signifying the importance of the corrosion type of metal loss and pressure. Few researchers employed the CBM approach, which involves monitoring the health status of a pipeline with sensors and some other diagnostic equipment to identify indications of corrosion, wear, and different types of deterioration, enabling predictive maintenance and life forecasting^13^. This research proposed standard principles for generating scenarios utilizing predicted pipeline conditions before and following maintenance measures. PRA, a risk-based strategy, was used by a few researchers who employed statistical models to forecast the chance of failure and assess the consequences of failure^14,15^. In these studies, one research created a tool that supports decisions to evaluate and manage the risk of oil and gas pipeline systems following an incidence of seismic events. Another study found that environmental variability and a lack of data are important sources of uncertainty about the effects of the oil spill. This demonstrates the importance of data management in evaluating the life of pipeline systems.

Many scholars have utilized ML and AI approaches to evaluate massive quantities of data and discover patterns that may be used to estimate the life of pipelines using historical data and real-time monitoring data^16–20^. These research works examined historical data from the oil and gas sectors, and the findings achieved by their different recommended methodologies were deemed satisfactory based on statistical metrics. In one of these studies, as much prior literature concentrated on one sort of failure, Senouci et al. used regression analysis (RA) and ANN to create a prediction model that considered factors apart from corrosion^18^. It was demonstrated that both strategies produced acceptable outcomes with similar accuracy in forecasting. In another study, their team created a fuzzy-based approach to anticipate the kind of failure in oil pipeline accidents. In this research study, they used many factors caused by mechanical, operating, corrosion, external, and natural risks to anticipate pipeline failures with a validity of 83%^19^. To improve the efficiency of these approaches, N B et al. employed Artificial Neural Networks (ANN) to forecast pipeline life conditions using historical data, and the findings of this study outperformed the previous approaches^17^. The relevance of these investigations lies in their ability to improve the capacity to anticipate pipeline conditions, suggesting an integrated approach that considers each approach's individual strengths. However, in these independent investigations, the models developed could only forecast the pipeline's life and could not diagnose metal loss issues. After a while, N B et al. tried an exciting approach to develop an intelligent model using the ANN, where the model estimated both objectives, i.e., pipeline life, and simultaneously classified the metal loss defects^16^. According to these research investigations, their ideas are more helpful in the oil and gas sectors for avoiding excessive inspection expenditures and planning the maintenance schedule.

Some studies used data-driven approaches to conduct risk analysis and predict the failure of pipelines. These research studies have significantly contributed to pipeline integrity and risk assessments using various AI-based methodologies^21–24^. Shahriar et al. used fuzzy logic in one of these works to calculate the likelihood of fundamental events in a fault tree and to predict the chance of output event effects^21^. This work investigates the interdependence of numerous aspects that may impact analysis findings, and fuzzy utility value is proposed to perform risk evaluation for pipelines utilizing the triple-bottom-line sustainable development criterion. Mohamed et al. conducted two studies that focused on predicting maximum pitting corrosion depth, adding insights to awareness of corrosion-related dangers in pipelines. In one study, they employed Support Vector Regression (SVR) to estimate the maximum pitting corrosion depth. The suggested models' efficiencies were compared to the standard SVR model, whose hyper-parameters are obtained by trial and error, on the one hand, and empirical models, on the other^22^. Three meta-heuristic techniques improve SVR's ability to identify these hyperparameters' best alternatives. Their results showed that the SVR-Firefly model outperformed every approach investigated in this study. Later, the same team members developed frameworks for forecasting maximum pitting corrosion depth utilizing statistical methods^23^. They evaluated the effectiveness of several AI algorithms and determined the relative value of various unknown input characteristics in predicting maximum pitting corrosion depth. Milad et al. suggested a Bayesian inference approach, which was used to precisely anticipate when the pipe would most likely collapse based on observed crack development readings and cycle records. This study employed finite element analysis for various fracture lengths and depths^24^. Based on a Bayesian inference study involving and excluding hyperparameters, the results showed that using hyperparameters improved accuracy, which emphasizes the relevance of hyperparameters. All of these studies can give useful information on pipelines' present condition and future functionality. These studies highlight the different and growing approaches used to improve oil and gas pipeline integrity and risk assessment.

Many statistical methodologies, such as Reliability-Based Analysis (RBA), Survival Analysis (SA), and Weibull Analysis (WA), were used to forecast the life of oil and gas pipelines. Using the RBA technique, Hou et al. predicted a pipeline's dependability and chance of failure while accounting for operating circumstances, environmental considerations, and material attributes^25^. The authors of the SA research, Mohmoud et al., utilized time-to-failure data to simulate the probability of failure over time while accounting for filtering and reduction^26^. Hall et al. employed the Weibull distribution to forecast the life of pipelines and other components in their WA research, considering the impacts of stress, wear, and other factors that might affect the rate of deterioration^27^. AI and ML are fast-evolving fields that have the potential to transform the way oil and gas pipelines are monitored, maintained, and operated. Previous research studies employed ML-based methodologies such as RA, ANNs, SVM, Random Forest (RF), and Decision Trees (DT) to forecast the life of oil and gas pipelines. Few researchers employed RA to forecast the remaining life of a pipeline using previous data such as inspection findings, maintenance records, and operating conditions^28^. In a few instances, ANNs beat RA in modelling complicated relationships and making predictions based on various input factors from historical data^17,18^. Few scholars employed SVMs and DTs to forecast oil and gas pipeline faults and risk management, respectively^29,30^. Ning et al. used the RF approach for the leak detection of gas pipelines based on multiple decision trees, resulting in more robust and trustworthy predictions by aggregating the findings of many trees, each of which may be trained on a different subset of the data^31^. These AI and ML-based algorithms provided useful insights concerning pipelines' present and future behaviour, allowing operators to prioritize maintenance and replacement actions to maintain safe and dependable operation. The investigations in the preceding literature for pipeline life forecasting depend on prior knowledge to build structural health measures and determine thresholds, thus remaining ineffective in the significant data era.

ANNs are computational models inspired by the structure and function of the human brain. They comprise linked nodes known as neurons that analyze and send data. Image classification, audio recognition, and natural language processing are just a few of the tasks in which ANNs are utilized. The artificial neuron is the fundamental building component of an ANN; it accepts information from other neurons, analyses it, and sends a signal to other neurons in the network^32^. The weights of the neuronal connections can be modified during training to maximize the network's performance. ANNs are learned by employing algorithms that modify the weights of neural connections based on the difference between the expected and actual output. The training procedure might be unsupervised or supervised. Artificial intelligence research is always looking for new methods to increase the performance and interpretability of ANNs.

Since its origins in the 1940s and 1950s, ANN architecture has evolved tremendously. Initially, simple feed-forward networks were proposed with information flowing just in one way, from input to output. These networks' capacity to analyze complicated data and reflect links between inputs and outputs was restricted^33^. Recurrent neural networks were introduced in the 1980s and 1990s, where information may flow in loops, and the network can keep a hidden state, allowing it to handle input sequences. This signified a substantial increase in neural networks' capacity to handle sequential input. Deep neural networks gained popularity in the late 2000s and early 2010s as massive datasets and strong GPUs enabled the training of several layers of neural networks^34^. In recent years, novel ANN designs that specialize in tackling specific difficulties have been created. Overall, the desire to tackle particular tasks more effectively and efficiently has propelled the development of ANN design, and new structures are constantly being invented and improved.

Bayesian Regularization (BR) is a strategy for reducing overfitting in ANNs. When a model overfits, it learns random noise or irregular oscillations in the training data rather than the underlying patterns^35^. As a result, performance on unknown data, for instance, the validate or test set, suffers. BR combats overfitting by adding existing knowledge about the network's weights during training. This is accomplished by considering the weights as explanatory variables with a prior distribution, like a Gaussian distribution, and modifying the distribution as new data becomes available during training. The preceding distribution encodes previous views about how the weights should appear, preventing them from becoming excessively severe and overfitting to the data. The term regularization in the BR technique measures the weight distribution's uncertainties or variance. Optimizing the goal function during training becomes an exchange between prediction performance and weight entropy^36^. This enables the model to compromise accurate data fitting and limit overfitting. BR may be used on several types of ANNs, including feed-forward and recurrent networks. The approach has been proven to increase ANN generalization performance on various tasks, and it has been employed in a wide range of applications, such as image classification, life prediction, audio detection, and natural language analysis^37^.

According to research studies and the present market of the oil and gas sector, a few activities need to be made to increase the life of oil and gas pipelines. Regular maintenance and inspections, leak detection systems, upgraded pipeline infrastructure, environmentally friendly practices in design, construction, and operation, novel strategies developed with research and development experts, and emergency response plans are some of the activities that could improve the lifespan of pipelines. However, a few issues must be addressed to extend the life of dry gas pipelines, such as corrosion and deterioration, maintenance schedule, environmental restrictions, cybersecurity, and so on. In addition, because of the expensive inspection costs, suitable data for pipeline lifespan evaluation studies are not readily available, as evidenced by literature studies. As a result, even if the data is lacking, a practical framework for accurate forecasts of the life situation with corrosion flaws needs to be established. As a result, this study aims to estimate the life and detect the dimension class metal loss type of dry gas pipeline systems, which would lead to better safety, reliability, and maintenance schedules. In other words, while the current literature on pipeline life prediction, risk assessment, and failure evaluation has made remarkable progress in using techniques based on machine learning-enhanced frameworks, still a research gap exists in the establishment of dynamic and adaptable methodologies to regularly update structural condition indicators, making sure more precise and up-to-date pipeline life estimating in a continuously shifting operational environment. Also, an efficient framework providing accurate estimates of the life situation, including metal loss anomaly detection types, must be established to improve safety, business, and ecosystem, even if missing data exists. Inspired by the literature, this work provides a novel approach for predicting the life conditions of a dry gas pipeline system and detecting the dimension class of metal loss simultaneously while utilizing BR-based ANNs with complete and incomplete inputs comprising missing input variables. As a result, the following are the paper's significant contributions:

- With comprehensive input data, the BRNN is developed to estimate the dry gas pipeline system's life condition and simultaneously detect the dimension class metal loss type.
- Sub-neural networks are developed to anticipate missing input variables. The pipeline life is then estimated based on the expected missing input data.
- The suggested BRNN-based approach's performance is examined in a few case studies where, at minimum, one input parameter and a maximum of three inputs are missing.
- The proposed BRNN framework identifies the pipeline life and dimension class interdependencies, demonstrating its adaptability and resilience in a real-world environment.

## Methodology

This research study performs various stages to construct a BR-based ANN model, including data analysis/preprocessing, selecting an ANN architecture, model training, model validation, and correlation analysis. All of these stages are detailed below.

### Data acquisition/analysis

The historical data was gathered from oil and gas fields and analyzed thoroughly. The dataset is selected, and data division is performed in the ratio 60:20:20, i.e., for training, testing, and validating the ANN by preprocessing it. In other words, the gathered data is divided into three sections such as training, validation, and testing datasets. The training dataset (60%) is employed to train the model, the Validation dataset (20%) is used to modify hyperparameters to avoid overfitting, and the Testing dataset (20%) is utilized to assess the model's generalization capability. The training set comprises 60% of the total collection to expose the model to a considerable quantity of data to learn underlying trends and relationships within the training data, modifying the variables to reduce the training loss. The validation set (20%) optimizes hyperparameters and avoids overfitting. During training, the model's performance on the validation set is assessed, and hyperparameters are adjusted to guarantee effective generalization without overfitting the training data. The testing set (20%) is kept separate and unused during the training and validation processes. It provides a fair evaluation of the model's performance on unseen data. This assists in determining how effectively the model generalizes to new, previously encountered cases. This data partition in a 60:20:20 ratio believes in an understanding of having enough data for training, validation, and testing, guaranteeing that the proposed model is well-trained and thoroughly assessed.

**Figure 1:**
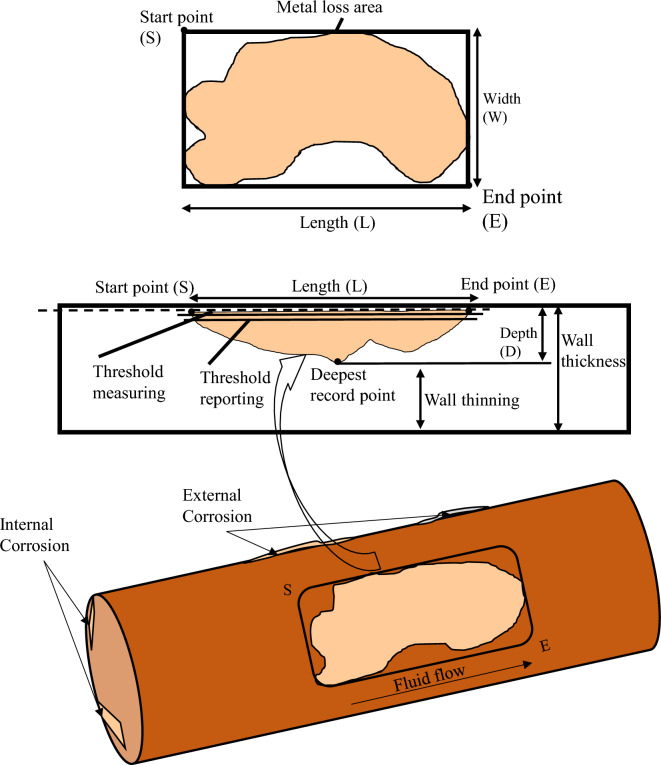
Pipeline features description.

During the data analysis, the parameters such as length (L), width (W), depth (D), nominal wall thickness (NWT), pressure (P), feature identification (FI), wall thinning (WT), and corrosion (C) are selected as inputs and parameters such as estimated repair factor (ERF) and dimension classification (DC) of metal loss are considered as outputs. All these input parameters are represented in Fig. 1 in detail. The dimensional parameters 'L', 'W', and 'D' are critical in interpreting metal loss measures since differences in length, width, and depth can obviously impact the pipeline's structural integrity. The variations from typical dimensions could indicate material abnormalities, leading to vulnerability. 'NWT' constitutes a standard measurement of the pipeline wall initiating thickness monitoring, critical for determining the pipeline's ability to endure operational pressures and external environmental conditions. The 'P' parameter heavily influences the structural stress encountered by the pipeline. Higher pressures may quicken material wear and tear, resulting in fatigue and potential failure. Monitoring pressure changes is critical for determining pipeline resilience and maintaining safe operation. 'FI' entails detecting particular traits or abnormalities in the pipeline. This might include material composition inconsistencies, structural abnormalities, or indications of probable degradation pathways. In general, FI refers to the process of detecting and classifying conventional signs (identifiable patterns) of physical objects, resulting in signaling within the gathered information. These signs indicate possible pipeline concerns or characteristics such as corrosion, manufacturing abnormalities, clustered corrosion, manufacturing anomalies, and mechanical irregularities. Corrosion, for example, may appear as inconsistencies in the thickness of the pipeline wall, whereas manufacturing anomalies may occur from flaws introduced during the production process. Clustered corrosion and manufacturing abnormalities suggest several cases in proximity, while mechanical anomalies may indicate external harm to the pipeline. A numerical indication (1–5) is created by assigning values for each of these recognised feature categories, allowing for better analysis and comparison of these characteristics throughout the whole dataset. The 'WT' of the pipeline is an indication of material loss from the pipeline walls. It is an essential measure for determining the level of damage that could put the gas pipeline's structural integrity at risk. Corrosion is an environmental phenomenon that causes pipeline material to deteriorate due to its interaction with the surrounding atmosphere, leading to structural degradation, durability loss, and changes in the appearance of the pipe material. Corrosion in gas pipelines may develop both inside and outside, posing substantial threats affecting the system's reliability and operation. Identifying the distinctions between interior and exterior corrosion is essential for developing efficient surveillance and safeguarding measures. Internal corrosion is the degradation of a pipeline's interior surface from chemical interactions between the delivered fluids and the pipeline's material. External corrosion happens on a pipeline's outside area and is usually impacted by environmental variables. Because there are multiple anomalies at various reference points throughout the pipeline system, Internal and external corrosion types are assigned values 1 and 2, respectively, in the current study.

**Figure 2:**
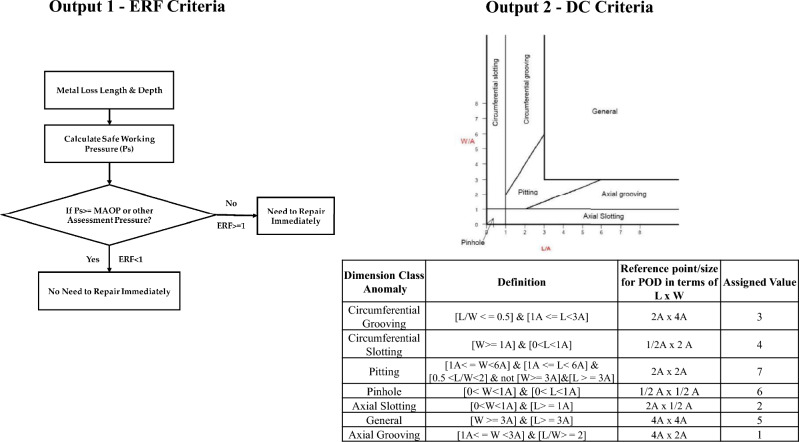
ERF and Metal loss DC criteria.

Every input parameter contributes to evaluating the pipeline's structural health, material attributes, and degradation processes. By including all these factors, it is possible to conduct a detailed examination that contributes to pipeline failure. Meanwhile, ERF denotes the ratio of the pipeline design pressure to the safe maximum pressure calculated as per the ASME B31G analytical criterion. According to pipeline integrity standards, ERF is used to evaluate the severity of pipeline life issues, and the ERF equation is provided by (1)^38^. In this relationship, the assessment pressure is the operator's pressure, and the safe operating pressure is the estimated defect assessment method's safe operation pressure. The choice of criterion to identify the level of severity of the pipeline condition was given according to the remaining durability evaluation technique, i.e., ASME B31G, as illustrated in Figure 2. DC refers to a distinct anomaly class allowing a wide range of metal loss forms. Figure 2 depicts the criteria for ERF and metal loss anomalies per dimension class. The best BRNN design is chosen for predicting the life dry gas pipeline, which is built with 8 input parameters, i.e. L, W, D, NWT, P, FI, WT, and C, with the two hidden layers and 10 neurons in each layer; and with two output parameters, i.e., ERF and D.

1 $$ ERF = \frac{{P_{assesment} }}{{P_{safe\;working} }} $$

Metal loss classification refers to classifying metal losses based on size and location. The purpose of metal loss categorization is to evaluate the degree and severity of corrosion in a metal structure so that necessary repairs or additional damage may be made. According to ASME B31G standards, metal losses are divided into various dimensions: pitting corrosion, general corrosion, axial grooving, circumferential grooving, pinhole, axial slotting, and circumferential slotting^16^. The pipeline operators forum (POF) has created a set of standards and criteria for intelligent pig inspections of pipelines, including descriptions of metal loss characteristics^39^. The severity of metal loss based on L, W, and D was used to classify the dimension class in the proposed study. All DC categories are assigned numerical values (1 to 7), and the criteria used to classify DC anomalies of metal loss are illustrated in Fig. 2. Notably, the order of numerical values is determined by the alphabetical listing of the DC of metal loss. In other words, the DC criteria were classified depending on the metal loss in each portion of the pipeline system. All of these dimension classes' descriptions included with assigned values are given in Fig. 2.

According to the data collected, the pipeline system is separated into four sections: Section 1, Section 2, Section 3, and Section 4, each having a length of 123.4 km, 225 km, 139.56 km, and 22.2 km. These four sections are all built of Spiral/Longitudinal welded pipe with an API 5L X70 material grade and a diameter of 36 inches. Section 1, Section 2, Section 3, and Section 4 have NWTs of 16.8/19.8/21.4 mm, 14.2/16.8/19.8/22.7 mm, 14.23/16.76/19.79 mm, and 16.76/19.79 mm, respectively. All of these pipeline system details are illustrated in Table 1.

**Table 1:** Pipeline system characteristics.

Feature	Section 1	Section 2	Section 3	Section 4
Length	123.4 km	225.0 km	139.6 km	22.2 km
Diameter	36 inches	36 inches	36 inches	36 inches
Material grade	API 5L X70	API 5L X70	API 5L X70	API 5L X70
Nominal wall thickness	16.8/19.8/21.4 mm	14.2/16.8/19.8/22.7 mm	14.23/16.8/19.8 mm	16.8/19.8 mm
Pipe type	Spiral/Longitudinal welded	Spiral/Longitudinal welded	Spiral/Longitudinal welded	Spiral/Longitudinal welded
Design code	ASME B31G	ASME B31G	ASME B31G	ASME B31G
MAOP	96 barg	96 barg	96 barg	96 barg
Design pressure	96 barg	96 barg	96 barg	96 barg
Pipeline product	Dry Gas	Dry Gas	Dry Gas	Dry Gas

Based on past inspection reports obtained during data gathering, A total of 5406 (5235 internal and 171 external), 4452 (4124 internal and 328 external), 1266 (1001 internal and 265 external), and 234 (213 internal and 21 external) metal loss abnormalities were found and recorded for Section 1, Section 2, Section 3, and Section 4 respectively. These recognized and recorded metal loss anomalies during the data analysis indicate that the pipeline has suffered metal loss degradation in many locations. Anomalies in metal loss indicate different types of corrosion, eroding, or other material deterioration that threaten the structural integrity of the pipeline system. The significant frequency of anomalies emphasizes the need to ensure the security and reliability of a dry gas pipeline's system. Analyzing these particular features and the distribution pattern of these anomalies is critical in developing and implementing strategies, reducing the risk of failure, and assuring efficient operation. All of these metal loss anomalies are illustrated in Table 2 in detail.

**Table 2:** Detected anomalies by depth and ERF threshold.

Depth	Section 1		Section 2		Section 3		Section 4	
	Internal	External	Internal	External	Internal	External	Internal	External
80% and above	–	–	–	–	–	–	–	–
70% ≤ metal loss < 80%	–	–	–	–	–	–	–	–
60% ≤ metal loss < 70%	–	–	–	–	–	–	–	–
50% ≤ metal loss < 60%	–	–	–	2	–	–	–	–
40% ≤ metal loss < 50%	–	–	–	–	–	–	–	–
30% ≤ metal loss < 40%	14	–	1	–	4	–	–	–
20% ≤ metal loss < 30%	233	2	31	3	10	–	10	–
10% ≤ metal loss < 20%	4988	169	4092	323	987	265	203	21
Total no. of anomalies	5235	171	4124	328	1001	265	213	21
ERF								
ERF < 0.6	105	2	–	–	6	0	40	–
0.6 ≤ ERF < 0.8	5130	169	1127	57	213	65	173	21
0.8 ≤ ERF < 1.0	–	–	2997	271	782	200	–	–
ERF > 1.0	–	–	–	–	–	–	–	–
Total no. of anomalies	5235	171	4124	328	1001	265	213	21

### BR neural network learning

This work employs the BR-based back propagation (BP) approach to create a suggested ANN model. The proposed technique combines the ideas of Bayesian inference with backpropagation to increase prediction accuracy^39^. It was initially developed as an extension of classic ANN models in the early 1990s. The proposed model employs BR to include previous information about the model's parameters and to minimize overfitting. This is accomplished by having a penalty term in the error function that penalizes complicated models^40^. The backpropagation technique then identifies the ideal settings by minimizing the error function. It has been demonstrated to outperform typical ANN models and has become a significant tool in machine-learning studies. In the current study, BR was chosen over traditional techniques such as Levenberg Marquardt or others because of the dual output of the present study, which focuses on forecasting the life of a pipeline based on ERF while also determining its dimension class. This dual-objective situation necessitates a deeper approach to model training and parameter estimation, which the suggested BR technique effectively addresses our problem successfully. Notably, the BR approach enables the proposed framework to identify the interdependencies among the pipeline life and dimension class, demonstrating its adaptability and resilience in a real-world environment.

The current study chooses an appropriate prior distribution for the network weights, such as a Gaussian distribution with a zero mean and a modest standard deviation. The 'trainbr' algorithm calculates the posterior distribution of weights given data and a prior distribution. After seeing the data, the posterior distribution encodes the uncertainties regarding the weights^41^. In other words, Bayes' theorem combines the prior distribution with the likelihood function to generate the posterior distribution over the model parameters. Combining the previous distribution and the likelihood function yields the posterior distribution^42^. The regularized target function is computed using the posterior distribution (See Eq. 2). The negative log posterior distribution is the regularized objective function, identical to minimizing the sum of the squared error and the regularization term. The regularization term is a penalty term that promotes the model to match the prior distribution over the model parameters rather than fitting the noise in the data.

2 $$ J\left( w \right) = \left[ {\left( \frac{1}{2} \right)*\left( {y - xw} \right)^{t} *\left( {y - xw} \right)} \right] + \left[ {\left( \frac{1}{2} \right)*\left( w \right)^{t} *\left( {\sum w} \right)^{ - 1} } \right] + c $$

where, $w$ is a weight vector of model parameters, $y$ is a vector of output variables, $x$ is a matrix of input variables, and $c$ is a constant.

The squared error between the anticipated and actual values of the output variable is represented by the first term in the objective function. The second term is the regularization term, which is proportional to the square of the model parameters' magnitude. The constant $c$ governs the trade-off between the two terms and is usually determined through cross-validation. The BR-based ML approach aims to determine the ideal weights that maximize the posterior distribution, which is proportional to the likelihood function multiplied by the prior distribution. The optimum weights may be discovered by enhancing the posterior distribution. The related weights (*w*) of a BRNN are adjusted based on the error rate achieved in the prior epoch. Furthermore, hidden neuron weights are modified to ensure that the ANN model is highly efficient. The suggested BRNN calculates the ultimate output of hidden and output layers, as shown in (3).

3 $$ y_{i} = \sum x_{i} w_{i} + b_{i} $$

where, ${x}_{i}$, ${w}_{i}$ and ${b}_{i}$ are the initial, weight, and bias values of a variable.

The model is then evaluated by making predictions on the testing dataset using the posterior distribution and assessing the model's performance. Metrics like MSE and R^2^ values are used in the evaluation process. The selection of hyperparameters is an integral part of developing and training neural networks because it influences the model's performance and adaptability^43^. This external selection to the model is not learned throughout training but substantially influences its behaviour. The learning rate, batch size, number of hidden layers, and number of neurons in each layer are a few instances of these hyperparameters. Slow convergence, overfitting, and poor generalization can result from a poorly designed collection of hyperparameters^44^. The objective is to establish a compromise between underfitting and overfitting so the model performs well on training and unobserved data. Finally, selecting hyperparameters with care is critical to developing neural networks that successfully capture the underlying patterns in data.

**Figure 3:**
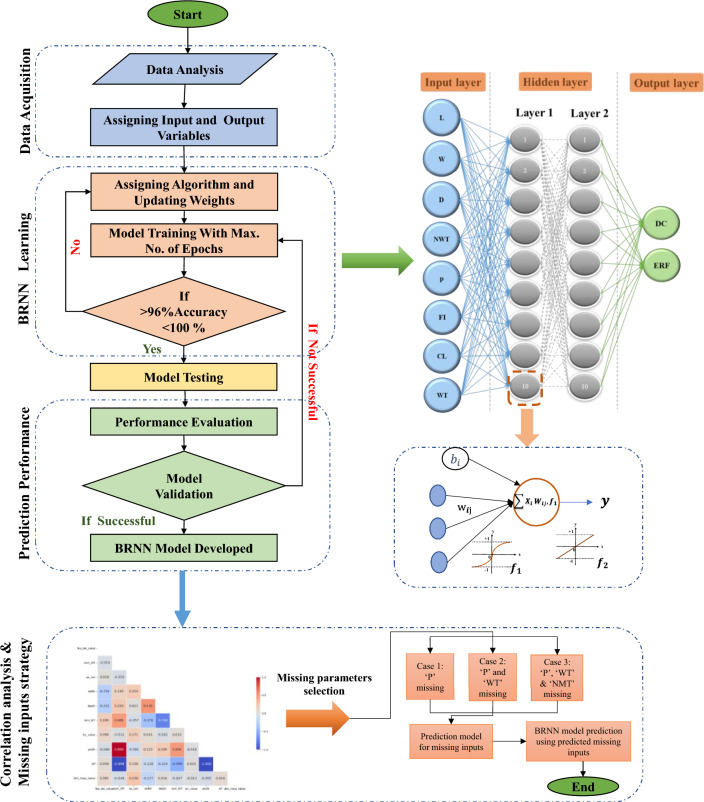
BR-based neural network framework.

The whole procedure of the proposed BR-based ANN framework is illustrated in Fig. 3. The network design, prior distribution, and training procedure are adjusted to increase the model's performance. Finally, the 'trainbr' (BR) based ANN with a topology of 8-10-10-2 provided the best performance with good evaluation performance, balancing the trade-off between accurately fitting the data well and avoiding computational burden. The activation functions such as 'tansig' and 'purelin' are used in hidden and output layers, respectively, as represented in Fig. 3. Implementing the BR-based ANN approach in this study is essential for limiting the possibility of overfitting. The BR technique presents a disciplined way of dealing with uncertainty in parameter estimations. This guarantees that the model generalizes adequately to previously unknown data points. In addition, BR provides a regularization term that prevents overfitting and allows the model to learn meaningful patterns while accounting for the underlying uncertainty in the data.

Figure 3 depicts the developed ANN model structure, which includes two hidden layers interposed between the input and output layers. The current study employs two hidden layers to learn complicated correlations between input and output data. As per Fig. 3 of an ANN model, the input layer receives the input data, which is transferred to the first hidden layer. This layer comprises neurons learning, processing, and forming intermediate approximations from the input data. These approximations are subsequently forwarded to the second hidden layer, where more processing and learning occur. Finally, the output layer generates the expected outcome depending on the information learned by the hidden layers. The purpose of using two hidden layers in this study is it has the benefit of being able to manage the complexity between input and output data. This implies the model may learn to detect patterns and generate more accurate predictions. Furthermore, it enables the model to learn and predict with greater depth and complexity, essential for handling increasingly difficult situations.

### Correlation analysis

Correlation analysis is a statistical technique for determining the relation between two or more variables. The correlation coefficient, which ranges from − 1 to + 1, determines the relationship's strength and direction. A correlation coefficient of − 1 shows an entirely negative correlation, which means that if one variable grows, so does not the other. A correlation coefficient of + 1 shows a complete positive correlation, which means that when one variable grows, so does the other. In correlation analysis, ANNs are frequently employed to find correlations between variables. One method for doing correlation analysis using ANNs has a single output node and several input nodes. The output node represents the variable of interest, while the input nodes represent the other variables being studied.

The ANN is trained on a series of input–output pairs, where the input values are the values of the other variables being evaluated and the output value is the value of the desired variable. Based on the input variables, the ANN learns to predict the output variable, and the weights may be used to detect the strength and direction of the correlations between parameters. Pearson's correlation matrix is used in this study to conduct a correlation analysis between the predictive parameters of the network and the dependent parameters^45^. In other words, Correlation analysis employing Pearson correlation on a developed BRNN is used in this study to examine the connection between the BRNN model's forecasts and the actual target values. The Pearson correlation coefficient, designated by the letter ‘${P}_{C}$’, measures the degree and direction of a linear link between two variables. A Pearson correlation matrix is a table that displays the correlation coefficients between distinct variables in a dataset^44^. The coefficients quantify the magnitude and direction of the linear relationship between two variables. The correlation coefficient between each pair of variables is then calculated using the following formula (4)^46^. This analysis is performed in the framework of a BRNN to analyze how well the model’s predictions agree with the real outcomes on a dataset different from that utilized for training, such as a validation or test set.

4 $$ P_{C} = \frac{{n\sum \left( {x*y} \right) - (\sum x)*(\sum y)}}{{\sqrt {n\sum x^{2} - (\sum x)^{2} } n\sum y^{2} - (\sum y)^{2} }} $$

where ‘${P}_{C}$’ is the correlation coefficient, x, and y are the values of the two variables, $\sum x$ and $\sum y$ are the means of the two variables. The interpretation of Pearson's correlation coefficients is discussed in the results section.

## Results and discussion

### Data analysis/visualization

**Figure 4:**
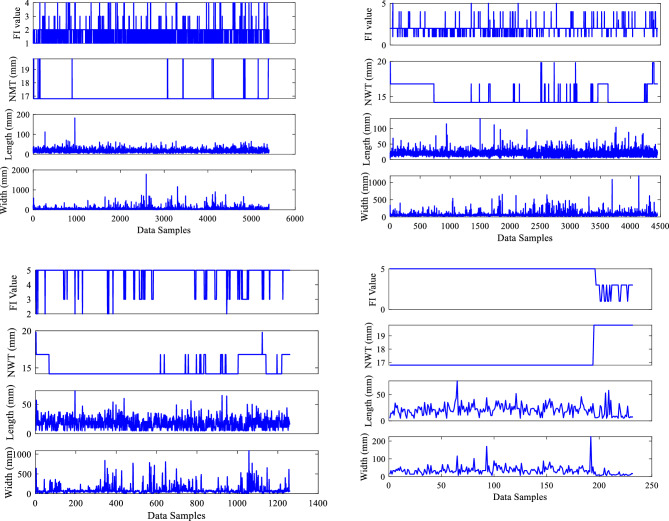
Inputs data visualization.

Data visualization is essential for displaying complicated data in visually appealing and comprehensible graphs. These visualizations reveal data insights that are difficult to obtain from raw data or written descriptions. Figure 4 represents an effective data visualization graph conveying information while portraying the underlying data quickly and effectively. Figure 4 shows that the X axis indicates data samples and the Y axis indicates the parameters of width (mm), length (mm), NWT (mm), and FI value with measurement units for the four sections of the dry gas pipeline system. Notably, the FI value is assigned in this study for the five separate categories recorded according to the POF, with matching allocated values: Corrosion (1), Cluster Corrosion (2), Cluster Manufacturing (3), Manufacturing (4), and mechanical (5). The generated data graphical representation plots successfully captured the distribution and trends of these discovered attributes. Figure 4 depicts each feature type, and their given values create a clear distinction, assisting in the visual examination. Also, the graphs depict the frequency and geographical distribution of five different types of FI throughout the dry gas pipeline.

**Figure 5:**
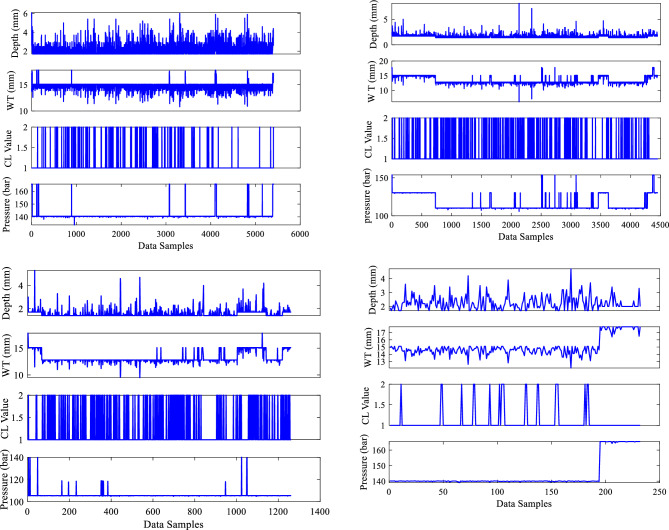
Inputs data visualization.

Figure 5 shows the data visualization plots with the X axis representing data samples and the Y axis representing the input parameters of depth (mm), WT (mm), corrosion location (CL) value, and pressure (bar) with units of measurement for the four sections of the dry gas pipeline system. Notably, in this study, the CL is divided into two categories: internal and external, with values 1 and 2 assigned to each. These two distinct classifications provide useful insights into corrosion trends via data visualization graphs, as shown in Fig. 5. The internal Corrosion graph with a value of 1 revealed distinct patterns and variations. The graph corresponding to exterior corrosion, indicated with the given value of 2, on the other hand, displayed distinct tendencies, allowing for a comparison of the two forms of corrosion. According to the acquired datasets, 5406, 4452, 1266, and 234 data sample values were recorded for these 8 input parameters for sections 1, 2, 3 and 4, respectively.

**Figure 6:**
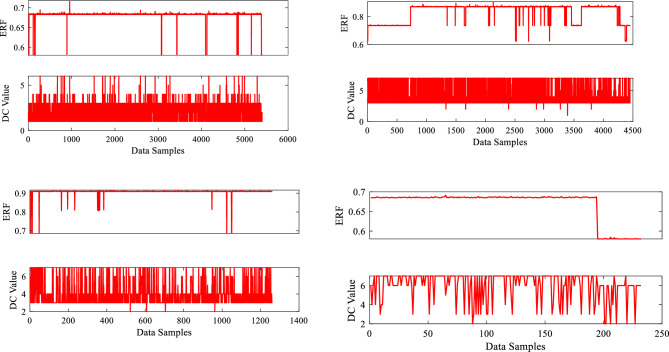
Outputs data visualization.

Similarly, the output parameters ERF and DC value are graphically represented using 5406, 4452, 1266, and 234 data sample values, with data points on the x-axis and output parameters on the y-axis. The metal loss DC data, which included seven types of abnormalities, is efficiently represented using a visualization with matching assigned values (1–7) in alphabetical order for all four sections of the dry gas pipeline. Each DC type has a unique numerical value, clearly depicting their relative importance. The visualization plot offers a distribution of these abnormalities, showing details regarding the frequency and extent of each category. The data visualization graphs for the two output variables evaluated in this study are shown in Fig. 6.

### BRNN modeling

**Figure 7:**
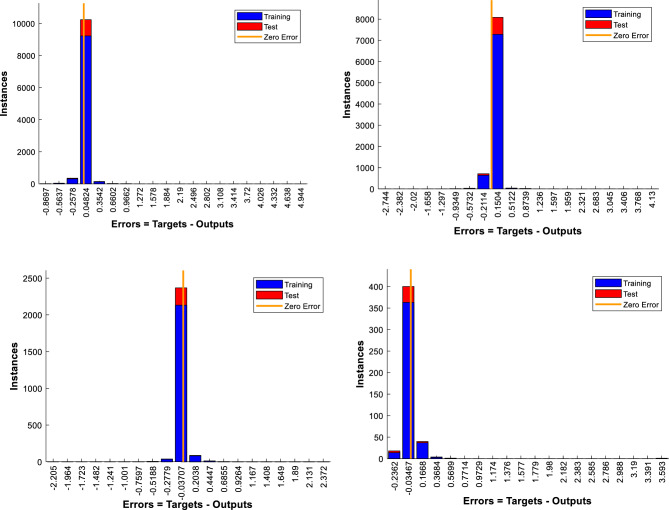
Error Histograms with 20 bins.

Error histograms depict the frequency of errors in values predicted by an ANN. These graphs aid in understanding how effectively the network performs and how faults are spread throughout the various classes or targets. An error histogram's x-axis normally shows the error range, while the y-axis reflects the frequency of mistakes within that range. If the histogram is broadly normal or uniform, it may imply that the mistakes are randomly dispersed and that the network could perform well overall. If the histogram, in contrast, side is distorted or has lengthy tails, it may suggest that the ANN is generating systematic errors. Figure 7 illustrates the error histograms recorded for the four training datasets of four sections.

**Figure 8:**
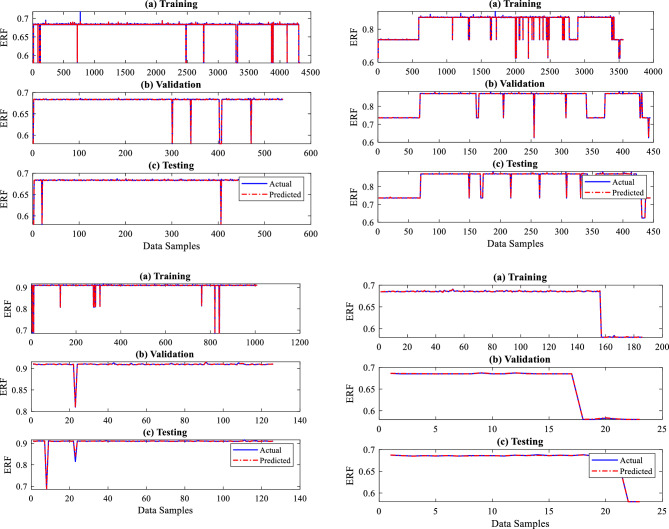
ERF prediction performance plots.

**Figure 9:**
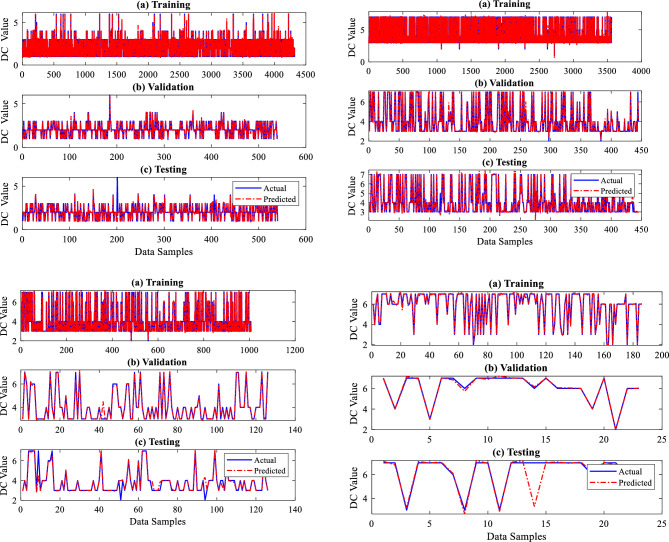
DC prediction performance plots.

Figures 8 and 9 illustrate the performance plots of four sections for the provided training, testing, and validation datasets, which are graphical representations of the performance of a developed BRNN. These serve to visualize the model's quality and progress as it is taught and assist in identifying potential patterns and issues during the training, testing, and validation process. Mean squared Error (MSE) is used in this study to quantify the change in the loss function of the ANN over time while it is being trained. The loss function measures how accurate the network is in making predictions. An increasing loss function shows that the ANN is overfitting or underfitting the data, whereas a decreasing loss function indicates that the network is improving. Accuracy is a measure of how well the ANN is making accurate predictions. The Coefficient of Determination (R^2^) and MSE values are used to identify how well the trained model is performed with the given datasets. From Fig. 8, the proposed BRNN predicted very well for a given dataset. The performance is checked using MSE, a loss function, and an R^2^. The MSE and R^2^ values are determined using Eqs. (5) and (6). The R^2^ and MSE values recorded during the training of BRNN are shown in Table 3.

5 $$ MSE = \frac{1}{n}\mathop \sum \limits_{1}^{n} \left( {y - y_{1} } \right)^{2} $$

6 $$ R^{2} = 1 - \frac{{\mathop \sum \nolimits_{1}^{n} \left( {y - y_{1} } \right)^{2} }}{{\mathop \sum \nolimits_{1}^{n} \left( {y_{mean} - y_{1} } \right)^{2} }} $$

where *n* is the number of data samples, $y$ is the actual, ${y}_{mean}$ is an average value and ${y}_{1}$ is predicted output.

**Table 3:** R^2^ and MSE values for all sections of the dry gas pipeline system.

Section	R^2^	MSE
Section 1	0.99449	0.000963
Section 2	0.99869	0.000964
Section 3	0.99811	0.000936
Section 4	0.99401	0.000955

The developed BR-based ANN model performed well in terms of dry gas pipeline life prediction. A robust prediction performance often depends on insightful graphs to give an understanding of the model's behaviour. Generating predicted performance plots for a given dataset during training is essential to evaluate the model's performance. Typically, validating a proposed BRNN model entails analyzing its capacity to generalize effectively to unknown data, which is critical for its real-world application. To capture the connections between the outputs in a model with two outputs, each output in this study, i.e., ERF and DC, are evaluated separately. The three-phase prediction performance plots, comparing anticipated outcomes to actual values, offer an easily understood visual representation of the predictive model's precision, as seen in Figs. 8 and 9. The predicted performance graphs from Fig. 8 show the expected values generated by the BR-based ANN compared to the actual values from the dataset. This provides a visual comparison of the model's performance. It can be seen clearly from Fig. 8 that ERF predicted values (dotted line) closely match the ERF actual values (continuous line), leading to a plot with a high-level connection between the two.

The developed BR-based ANN model performed well in dimension categorization prediction as well. Figure 9 illustrates the predicted performance graphs generated by the BR-based ANN comparing the expected DC values to the actual DC values from the dataset. Figure 9 shows the model's performance graphically well, as it can be seen that DC anticipated/predicted values (dotted line) closely matched with DC actual values (continuous line), resulting in a greater prediction performance by the proposed BRNN model. As per the results, the developed BRANN estimated life conditions and detected the DC metal loss type, which may assist pipeline engineers in determining the real situation and taking steps to enhance pipeline system reliability and safety. Therefore, a thorough examination of reliable statistical measures and informative graphs employed to construct a successful BRNN model with 10 inputs and two outputs provided an accurate understanding of the model's capabilities, assuring its dependability and efficiency.

### Correlation analysis

**Figure 10:**
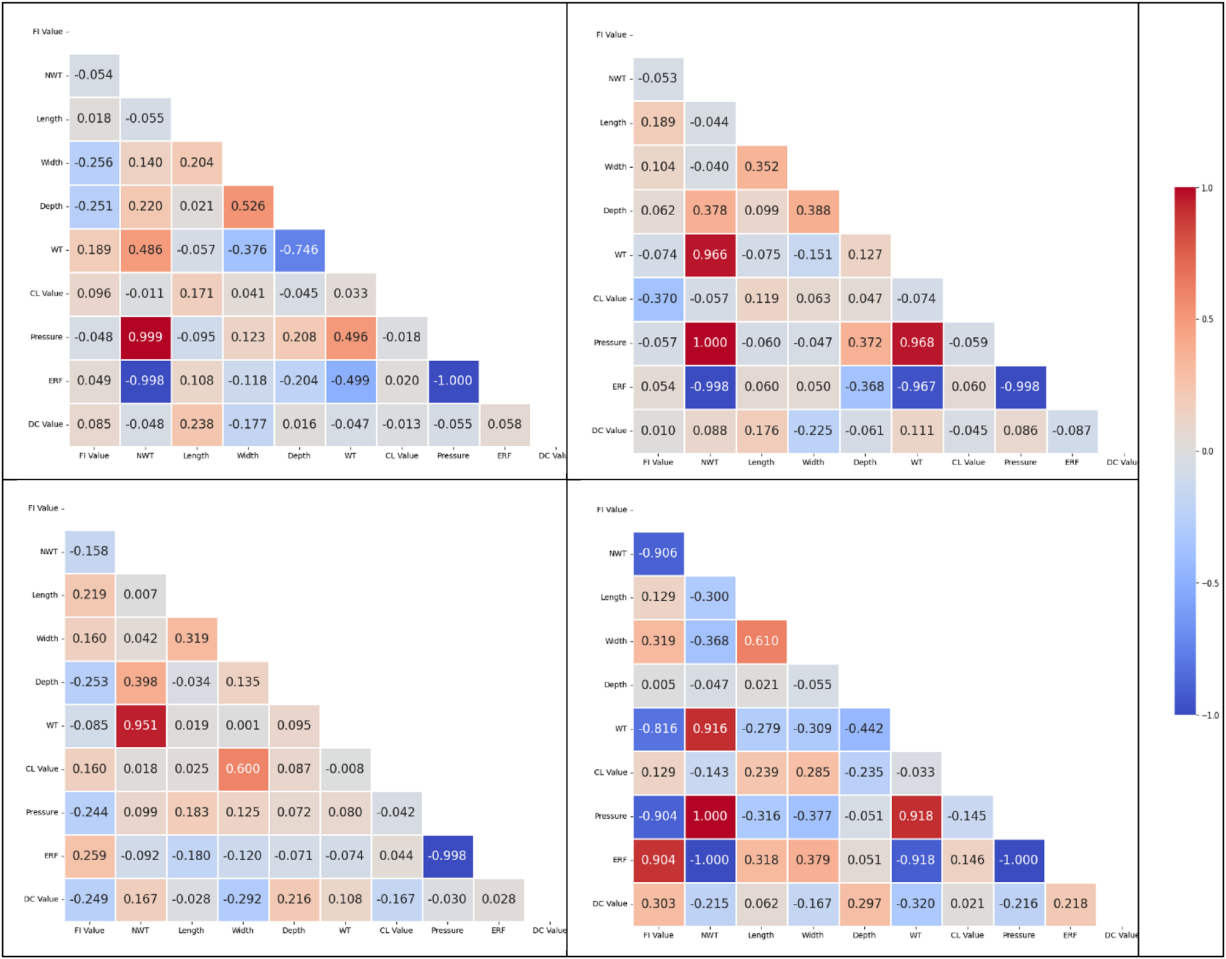
Correlation matrix for four sections.

Figure 10 shows the correlation matrix resulting in a square table with the same number of rows and columns as the data set's variables. The matrix diagonal depicts the correlation between each variable and itself, always equal to 1. Off-diagonal factors depict the relationship between each pair of variables. If the correlation is positive, the two parameters tend to rise or fall together; if the correlation is negative, the two parameters tend to move in opposing ways. Table 4 illustrates the interpretation of correlation coefficients ranging from ± 0.10 to ± 1.00. This correlation matrix is used for probabilistic reasoning about variable correlations or to estimate the parameters of proposed models with considered factors in this study.

**Table 4:** Pearson’s correlation coefficients interpretation.

Coefficient	Interpretation
± 0.90– ± 1.00	Very strong
± 0.70– ± 0.89	Strong
± 0.40– ± 0.69	Moderate
± 0.10– ± 0.39	Weak
0.00– ± 0.09	Negligible

During the correlation study, pressure, wall thickness, and wall thinning are important factors, highly correlated to pipeline deterioration among the eight variables investigated. The Pearson correlation coefficient, which is a statistical indicator of the connections between the two factors, is utilized to assess the intensity and direction of relationships between these parameters. This research discovered a substantial positive association between pressure and structural health failure, indicating that greater pressure rates were linked with a higher risk of dry gas pipeline collapse. Similarly, a negative association between NWT and pipeline failure is discovered, showing that walls with less NWT are correlated with a larger chance of structural difficulties. In other words, NWT was shown to be inversely related to pipeline failure, indicating that greater wall thickness leads to improved pipeline integrity. Furthermore, the correlation analysis revealed a significant negative connection with WT, implying that increasing thinning was associated with a higher probability of pipeline collapse. These discoveries provide light on the correlations between pressure, wall thickness, and wall thinning, laying out the selection basis for the missing input approach.

### Sensitivity analysis

Sensitivity analysis is essential in determining the corresponding effects of various characteristics on the outcomes, as well as identifying critical factors that have an important impact on the forecasts. Weight-based sensitivity analysis is a popular technique that entails assessing the effect of each weight in ANN. By finding and examining these significant weights, more about which factors have a greater impact on pipeline lifespan predictions can be learned. Using sensitivity coefficients allows one to statistically measure how sensitive the model is to alterations in every input characteristic. A larger sensitivity coefficient implies that specific parameters have a stronger effect on the result of the model. This quantitative examination of the influence of various variables improves the framework's interpretability while providing valuable insights for building focused preventative strategies. Recognizing which input characteristics have a highly significant impact on pipeline life estimate enables improved budget decision-making processes. Table 5 illustrates the sensitivity coefficients, which provide information about the influence of changes in the input variables on the proposed model's output.

**Table 5:** Sensitivity coefficients for four sections of pipeline system.

Factor	Section 1	Section 2	Section 3	Section 4
FI Value	1.6216	1.5117	14.0737	19.1700
NWT	33.3238	28.0769	5.0045	21.2032
Length	3.5969	1.6771	9.8155	6.7498
Width	3.9539	1.4138	6.5307	8.0369
Depth	6.8104	10.3434	3.8856	1.0775
WT	16.6519	27.2030	4.0040	19.4635
CL Value	0.6612	1.6917	2.3690	3.0913
Pressure	33.3804	28.0823	54.3170	21.2078

**Figure 11:**
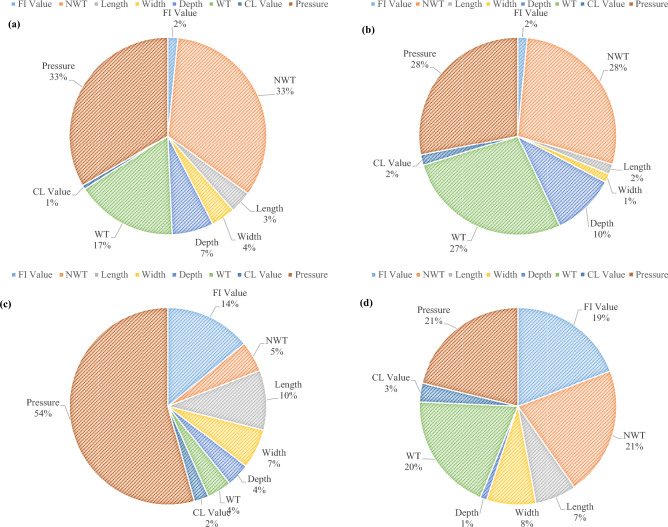
Relative contribution factors on pipeline life span.

In instances when the input variables have been missing, sensitivity analysis utilizing input variable fluctuation is useful. This strategy entails methodically altering input variables and tracking the resulting alterations in model forecasts. Evaluating the model's potential for disruptions offers beneficial details regarding the robustness of the lifetime estimation to changes in the input variables. In this study, the sensitivity coefficients determined by a sensitivity analysis on a BRANN model intended for dry gas pipeline life forecasting indicated that pressure, WT, and NWT have consistently greater values when compared with other variables throughout all four sections of the pipeline system, as presented in Table 5. This implies that variations in each feature have a greater impact on the estimated pipeline life. Therefore, missing input solutions have been implemented, focusing on these three factors to improve the overall dependability. Figure 11 depicts the distribution of sensitivity coefficients, highlighting the relative relevance of various input factors influencing pipeline life. The portion of every coefficient clearly visually represents variable relevance graphically.

In other words, these three variables were chosen based on their high sensitivity coefficients, showing that they significantly influence the precision of the ANN model for predicting pipeline life. In the lack of complete data, concentrating on these crucial factors reduces uncertainty in making predictions. When input data is unavailable, the sensitivity-driven attention to pressure, WT, and NWT helps decision-makers allocate resources for appropriate surveillance and inspection activities. Employing specific repair and administration alternatives to handle the increased sensitivity of these inputs results in a more durable approach to pipeline integrity.

### Sub neural networks development: handling missing inputs

This section creates and tests a novel prediction model for missing input parameters. The current study takes three missing input scenarios, which are discussed in the subsections Case 1, Case 2, and Case 3. The data is evaluated and pre-processed, and the missing inputs are defined in these scenarios. Three prediction models are developed using single-layered BRNN sub-neural networks to address the missing inputs in the three scenarios. The primary BRNN is trained and evaluated after the missing input parameters are anticipated to forecast the pipeline life condition (ERF) and detect each case's metal loss type (DC). All three cases with chosen missing input parameters are discussed below.

#### Case 1: One input—pressure (P) missing

**Figure 12:**
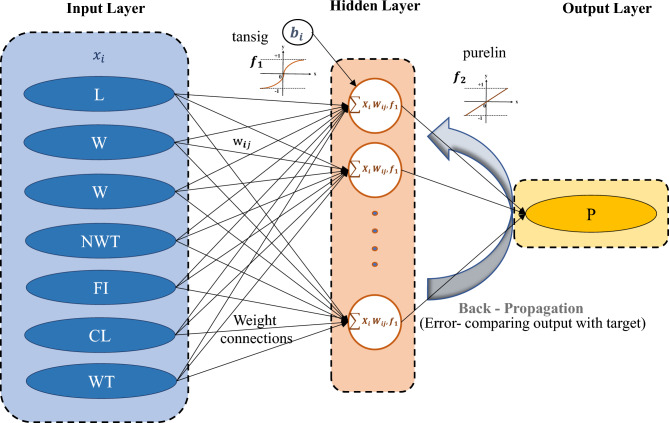
Sub-neural network to predict the missing variable pressure (P).

In the first scenario, one parameter, i.e., pressure, was chosen since it has a strong relationship with the contribution to the deterioration of the dry gas pipeline system. The pressure for the four dry gas pipelines is predicted using a BR-based sub-neural network (BRNN). The datasets utilized in this instance include 5406 samples from section 1, 4452 samples from section 2, 1266 samples from section 3, 234 samples from section 4. Figure 12 depicts a single-layered BRNN used to forecast pressure based on input data. The first layer in the architecture represents the input layer, which consists of 7 nodes that correspond to the 7 input parameters L, W, D, NMT, WT, CL value, and FI value. The single hidden layer nodes comprise 10 nodes, and the prediction model employs ‘tansig’ and ‘purelin’ activation functions, as shown in Fig. 12. The third layer represents the variable pressure that is missing.

**Figure 13:**
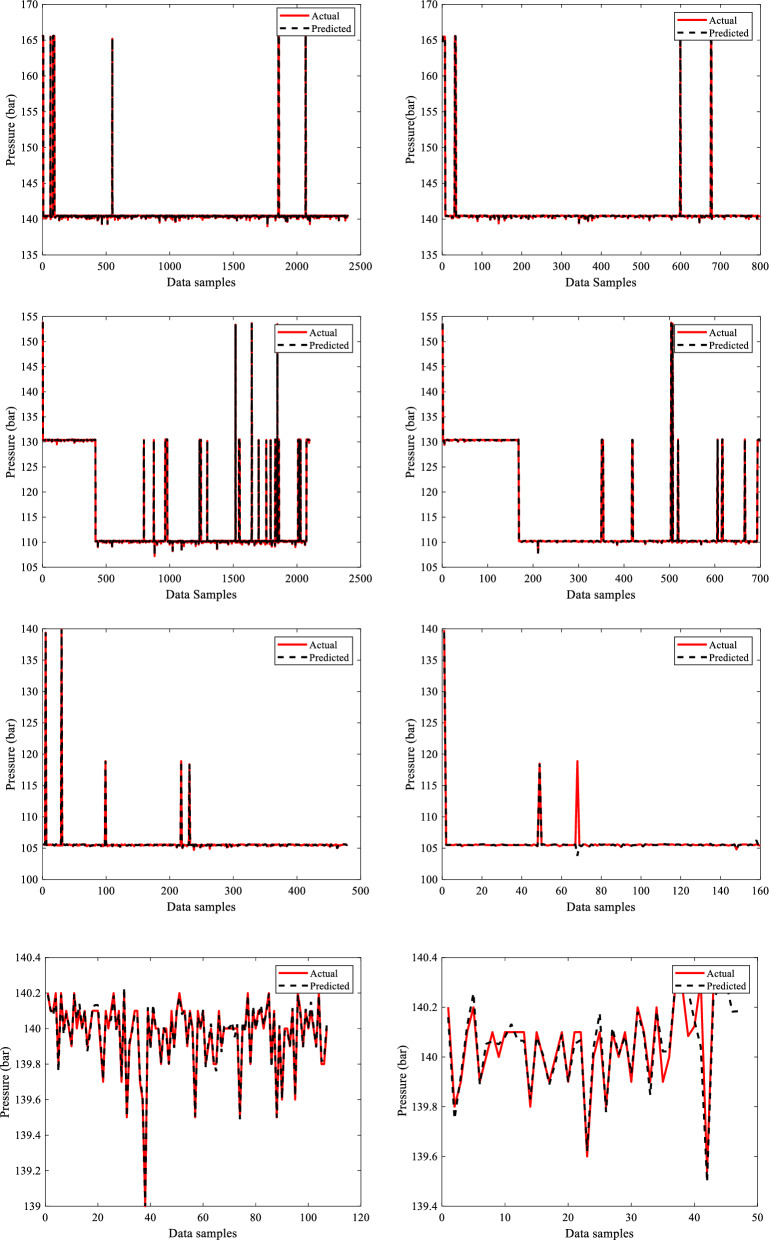
Training and testing performance plots for the missing pressure (P).

**Figure 14:**
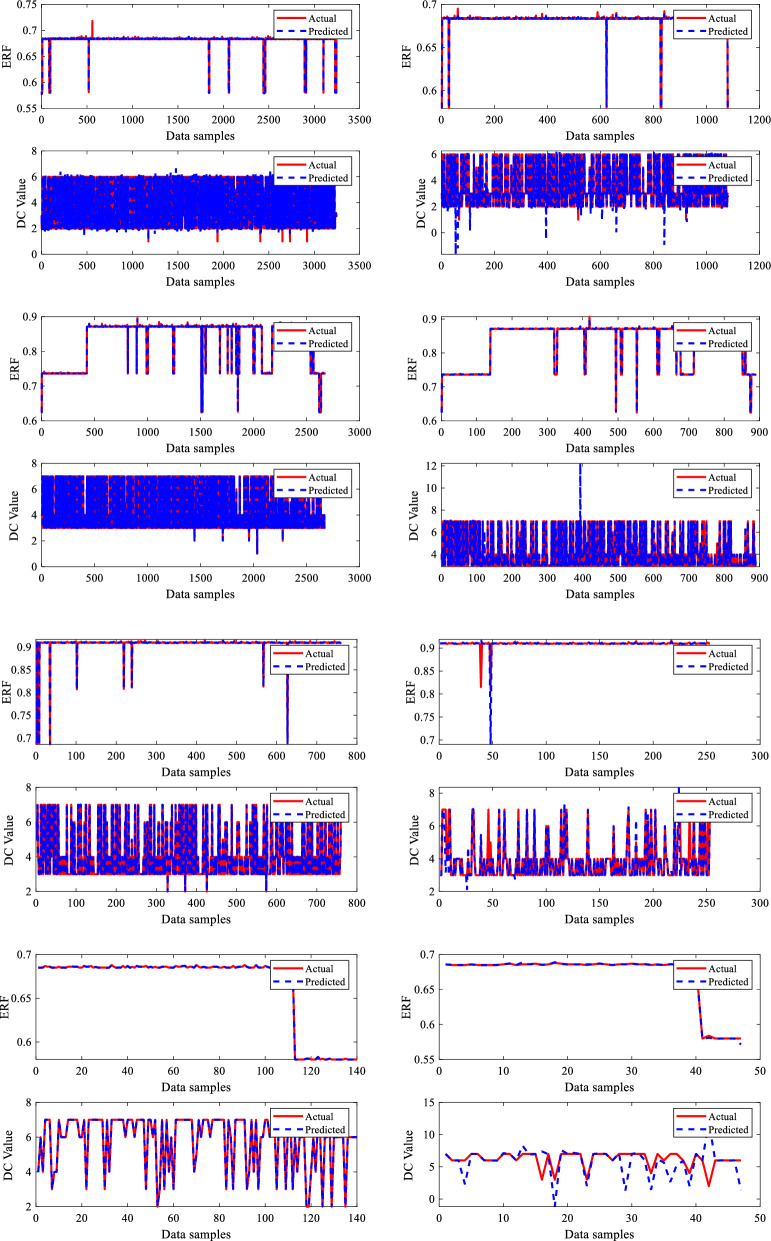
Training and testing performance plots of primary BRNN for case 1.

Figure 13 depicts the training and testing performance plots for all four dry gas pipeline system sections for the missing pressure scenario produced by the developed sub-neural network for the provided data samples. The performance of the sub-neural network is evaluated using R^2^ and MSE. The models acquired overall R^2^ values of 0.99995, 0.99997, 0.97513, and 0.9880 and MSE values of 0.00083, 0.00236, 0.000626, and 0.00963 for sections 1, 2, 3 and 4 respectively. The summary of all these values is given in Table 6. The primary BRNN is given a dataset that is replaced with the predicted missing input variable P, and it is trained and tested to predict outputs such as ERF and DC. Figure 14 illustrates the training and testing findings for case 1 by the principal BRNN for given data. The models obtained overall R^2^ values of 0.996, 0.9987, 0.99363, 0.96867 and MSE values of 0.00659, 0.00456, 0.00756 and 0.0387 for sections 1, 2, 3 and 4, respectively.

**Table 6:** MSE and R^2^ values recorded for sub-neural networks and primary neural networks.

Missing input	Section	Network	R^2^	MSE
P	Section 1	Sub neural network	0.99995	0.00083
		Primary neural network	0.996	0.00659
	Section 2	Sub neural network	0.99997	0.00236
		Primary neural network	0.9987	0.00456
	Section 3	Sub neural network	0.97513	0.000626
		Primary neural network	0.99363	0.00756
	Section 4	Sub neural network	0.9880	0.00963
		Primary neural network	0.96867	0.0387
P and WT	Section 1	Sub neural network	0.99999	0.000389
		Primary neural network	0.97499	0.02687
	Section 2	Sub neural network	0.99997	0.000408
		Primary neural network	0.97317	0.04897
	Section 3	Sub neural network	0.99987	0.000262
		Primary neural network	0.97372	0.0687
	Section 4	Sub neural network	0.99795	0.007366
		Primary neural network	0.97985	0.08657
P, WT and NWT	Section 1	Sub neural network	0.9997	0.000642
		Primary neural network	0.9937	0.00468
	Section 2	Sub neural network	0.99714	0.0005987
		Primary neural network	0.98593	0.02654
	Section 3	Sub neural network	0.9998	0.000692
		Primary neural network	0.98695	0.06587
	Section 4	Sub neural network	0.99972	0.00714
		Primary neural network	0.98182	0.06245

#### Case 2: Two inputs—pressure (P) and wall thinning (WT) missing

**Figure 15:**
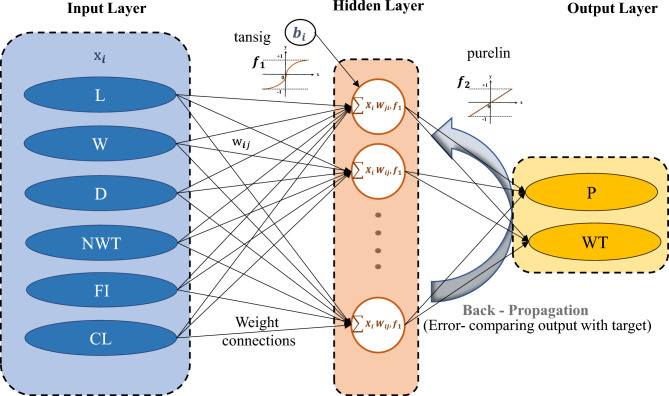
Sub-neural network to predict the missing Pressure (P) and Wall Thining (WT).

The two input parameters, i.e. P and WT, were chosen in the second scenario because they significantly correlate with the other factors' contribution to the degradation of the dry gas pipeline system. In this case, a BR-based sub-neural network (BRNN) is used to estimate the pressure and WT for the four dry gas pipelines. The dataset sizes used in this case are identical to those used in the previous scenario (case 1), namely 5406, 4452, 1266, and 234 samples from sections 1, 2, 3 and 4, respectively, but with two missing outputs (P and WT) this time. Figure 15 shows a single-layered BRNN forecasting P and WT based on a given dataset. The first layer in the design represents the input layer, which comprises six nodes corresponding to the six input parameters L, W, D, NMT, CL value, and FI value. The hidden layer nodes comprise 10 nodes, and the prediction model employs 'tansig' and 'purelin' activation functions, as shown in Fig. 15. The third layer represents the variables P and WT that are missing.

**Figure 16:**
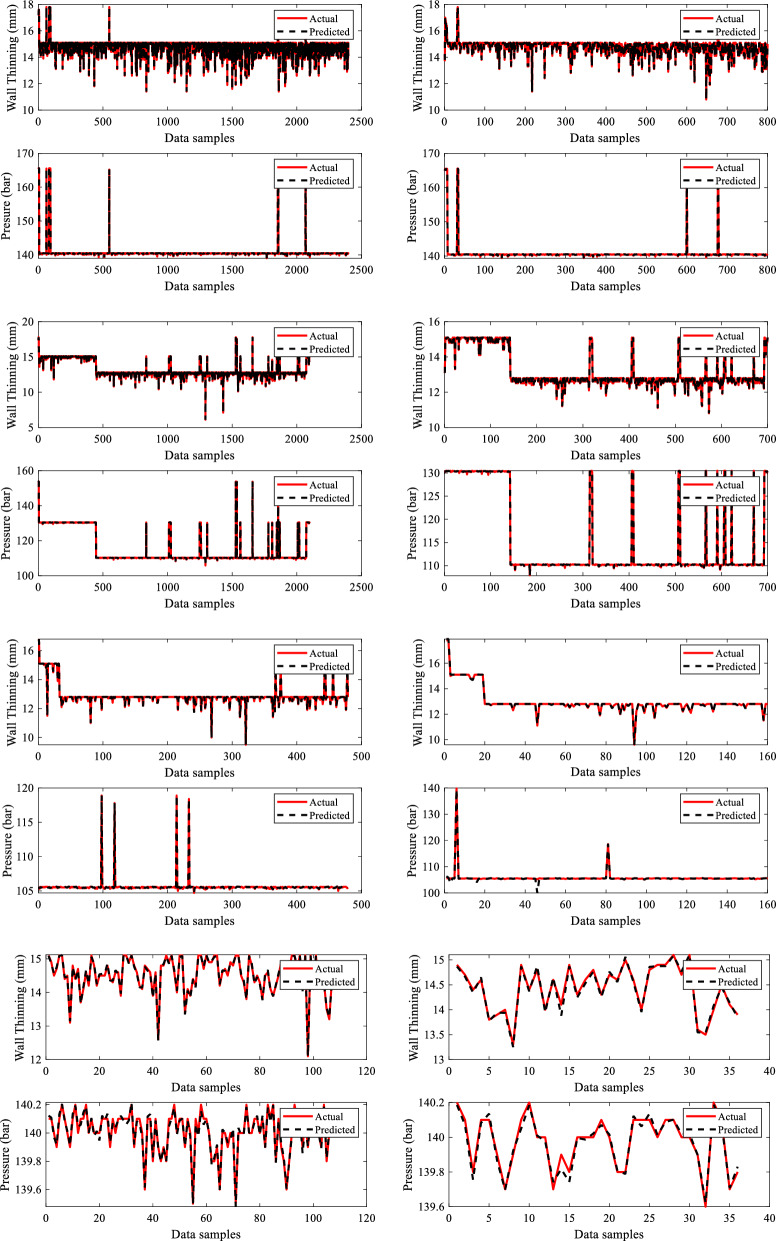
Training and testing performance plots for missing Pressure (P) and Wall Thining (WT).

**Figure 17:**
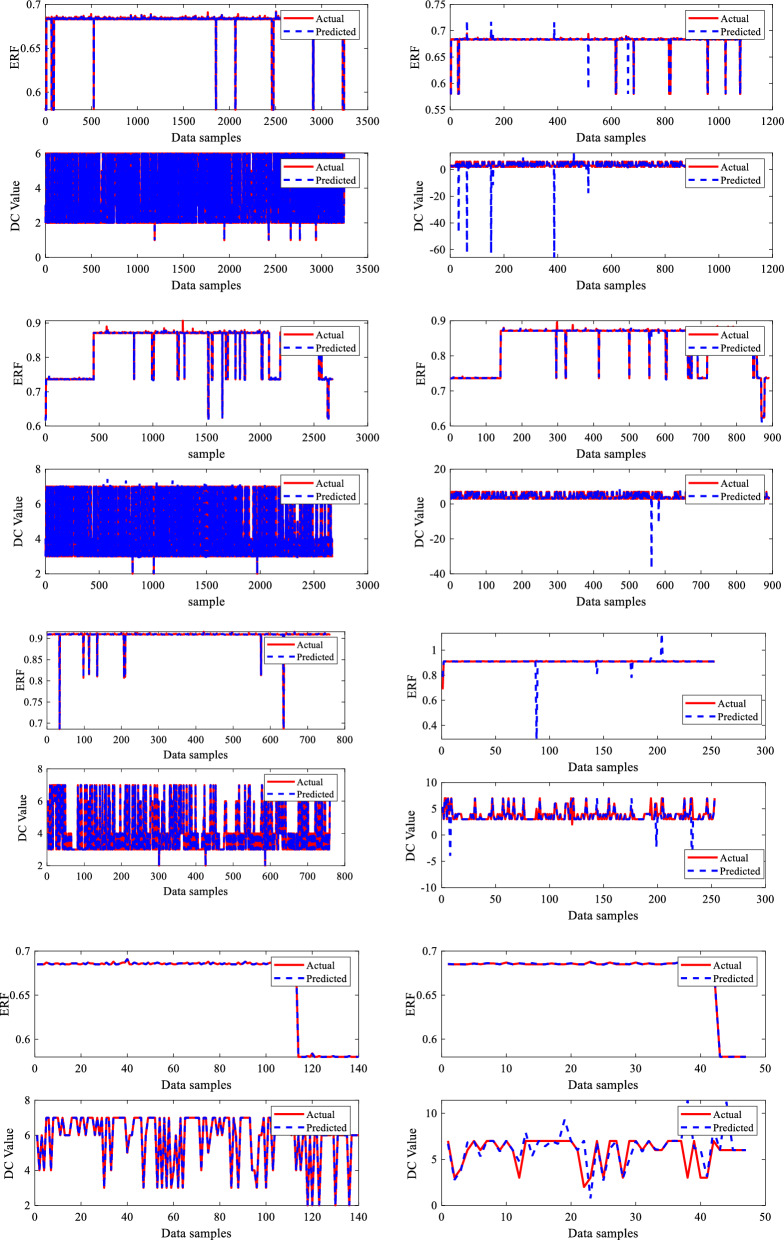
Training and testing performance plots of primary BRNN for case 2.

Figure 16 demonstrates the training and testing performance of the built sub-neural network for the missing P and WT situation using the given data samples. R^2^ and MSE are used to assess the performance of a sub-neural network. The models achieved overall R^2^ values of 0.99999, 0.99997, 0.99987, and 0.99795 and MSE values of 0.000389, 0.000408, 0.000262, and 0.007366 for sections 1, 2, 3 and 4 respectively. The summary of all these values for the four sections is given in Table 6. The primary BRNN is trained and evaluated to predict outputs such as ERF and DC using a dataset substituted with the estimated missing input variables P and WT. Figure 17 depicts the primary BRNN's training and testing results with the provided datasets. The BRNN performed well based on the R^2^ values of 0.97499, 0.97317, 0.97372 & 0.97985 and MSE values of 0.02687, 0.04897, 0.0687 and 0.08657 for sections 1, 2, 3 and 4 respectively.

#### Case 3: Three inputs—pressure (P), wall thining (WT), and nominal wall thickness (NWT) missing

**Figure 18:**
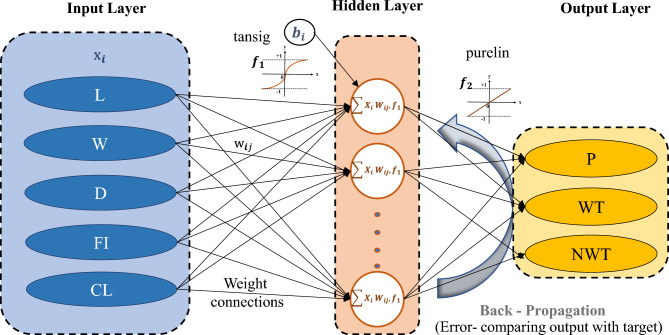
Sub-neural network to predict the missing Pressure (P) Wall Thinning (WT), and Nominal Wall Thickness (NWT).

In the third scenario, the three inputs i.e. P, WT, and NWT were chosen because they are the top variables that have a substantial correlation with the contribution of the other factors to the degradation of the dry gas pipeline system. In this case, too, a BR-based sub-neural network (BRNN) is utilized to estimate the P, WT, and NMT for the four dry gas pipelines. The dataset sizes utilized in this scenario are the same as those used in the previous two situations (case 1 and case 2) but with a different number of inputs and outputs (3 missing outputs P, WT, and NMT). A single-layered BRNN predicting P, WT, and NMT for a given dataset is shown in Fig. 18. The input layer is made up of five nodes, which correspond to the five input parameters L, W, D, CL value, and FI value. Whereas the hidden layer and output layer are made up of ten nodes and 3 nodes.

**Figure 19:**
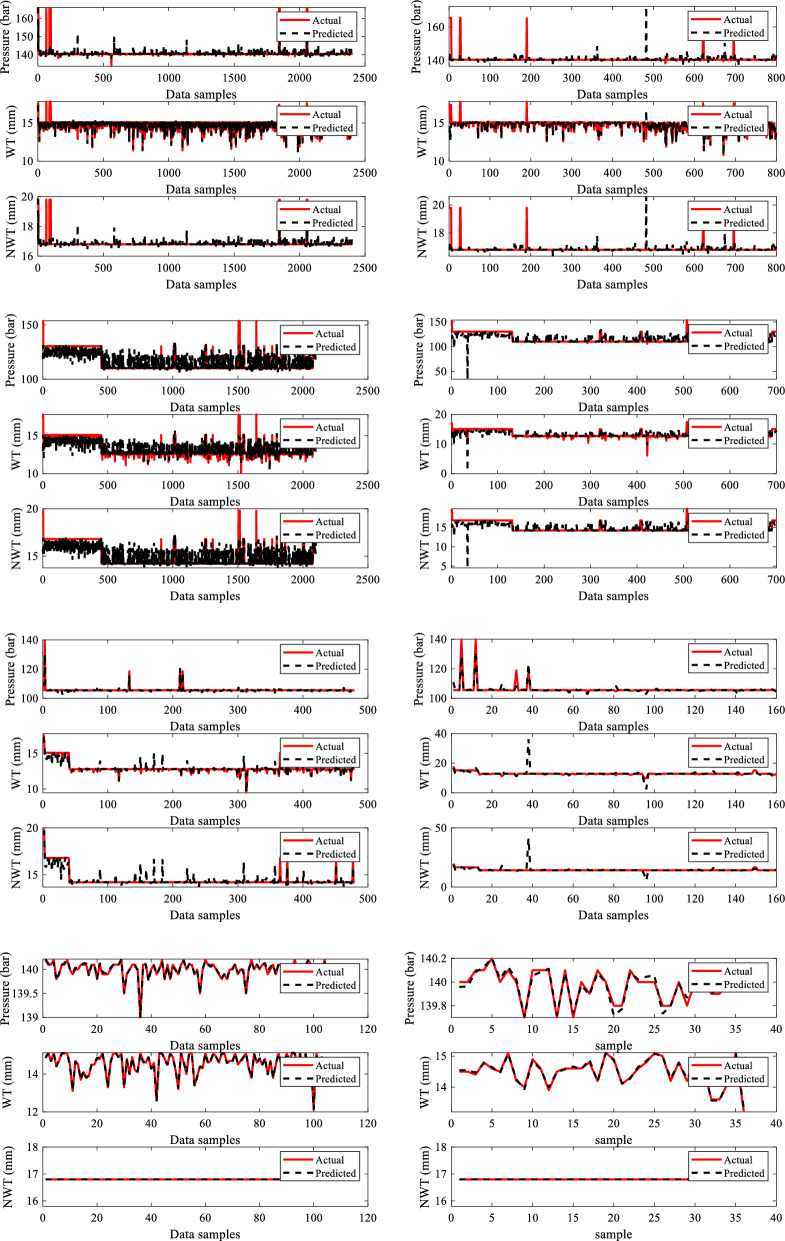
Training and testing performance plots for missing Pressure (P) Wall Thinning (WT), and Nominal Wall Thickness (NWT).

**Figure 20:**
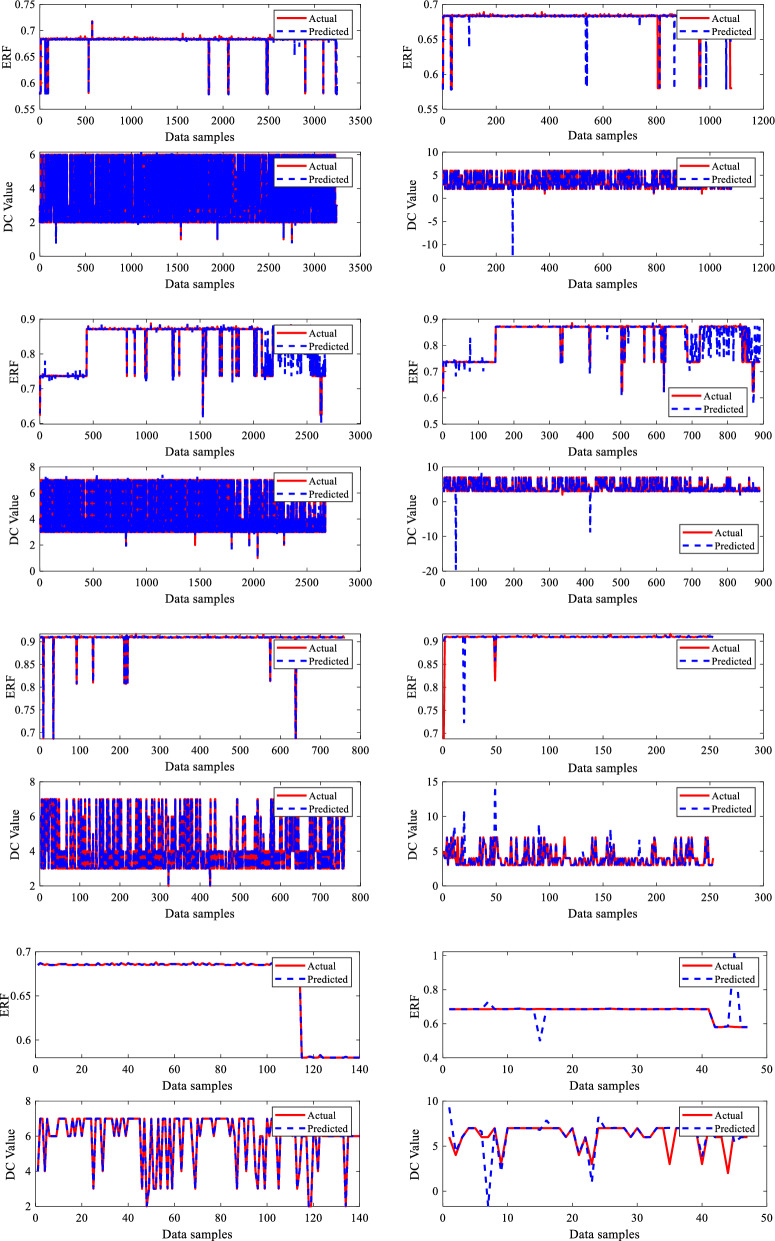
Training and testing performance plots of primary BRNN for case 3.

Figure 19 shows the training and testing performance graphically for the created sub-neural network on the supplied dataset for the missing P, WT, and NMT. The models performed well as they achieved overall R^2^ values of 0.9997, 99,714, 0.9998, and 0.99972 and MSE values of 0.000642, 0.0005987, 0.000692, and 0.00714 for sections 1, 2, 3, and 4 respectively as given in Table 6. Using a dataset that has been substituted with the predicted missing input variables P, WT, and NWT, the main BRNN has been trained and assessed to predict outputs such as ERF and DC for the four sections of the dry gas pipeline system. Figure 20 displays the training and testing outcomes of the principal BRNN using the given datasets for the four dry gas pipelines. Based on the overall R^2^ values of 0.9937, 0.98593, 0.98695 & 0.98182, and the MSE values of 0.00468, 0.02654, 0.06587 & 0.06245, for sections 1, 2, 3, and 4 respectively, the BRNN performed well.

The results obtained from the above scenarios indicate that the created framework would be beneficial in predicting life situations and DC metal loss types of pipelines even when data is missing. Thus, by evaluating historical data and determining the correlation between the parameters, this method could be used to forecast and monitor pipeline system behaviour, resulting in better reliability and safety. In other words, the probabilistic outputs offered by the proposed BR-based ANN in predicting the life of a dry gas pipeline and DC, which has critical uncertainties in the study, provided a more precise and accurate depiction of prediction uncertainty.

In the future, there may be difficulties in gathering and recording relevant data and the challenges suggested by the working environment of oil and gas pipelines, which are likely to be common problems in pipeline management and integrity evaluation. Several aspects, such as having historical data reports obtained using sensors for monitoring systems with built-in error-checking methods and utilizing powerful machine learning algorithms to discover abnormalities and outliers in data, are advised to address these concerns. However, because the missing input strategy is used in this study, the suggested BRNN framework can still adapt to changes in the operating environment and accommodate data uncertainties in real-time applications.

### Multi-model comparative analysis

**Figure 21:**
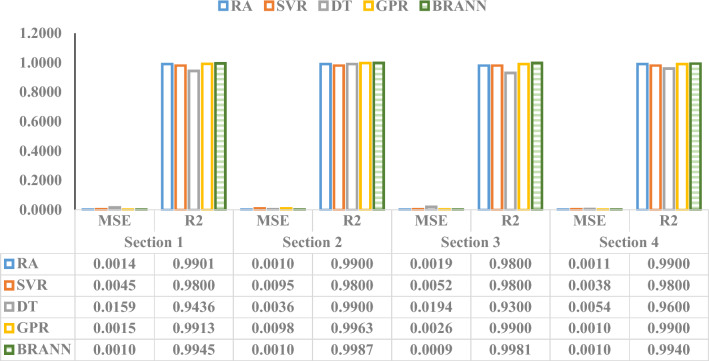
Numerical performance comparison of multiple ML-based techniques in predicting the target value.

Additionally, a comparative analysis has been conducted in this study. Employing a multi-model comparative analysis, which incorporates several ML-based algorithms such as RA, SVR, DT, and GPR, gives a complete strategy for evaluating the performance of various algorithms in predicting pipeline life. This comparison study provides a full assessment of multiple models' strengths and limitations based on statistical metrics, making it easier to choose the best technique for better reliable pipeline life prediction. While evaluating the abilities of different frameworks, it is critical to consider several measures, such as accuracy and computing efficiency. Every approach may have unique properties that affect its appropriateness for pipeline life estimations. Analyzing error outcomes across different approaches helps comprehend their strengths and shortcomings. While a model may thrive in certain areas, it may suffer in others, like generalization to unknown data or resilience to missing data. During a thorough comparison in this investigation, the notable variations in outcomes between the prediction algorithms have been recorded, as illustrated in Fig. 21. The analysis of multiple ML-based techniques reveals that BRANN constantly outperforms other approaches, demonstrating noteworthy accuracy in predicting the target value. BRANN is the best-performing algorithm in this analysis, with continuously lower MSE and higher R^2^ values throughout all sections. This outstanding outcome demonstrates BRANN's resilience and reliability in collecting complicated patterns in data and making correct predictions. Given its higher efficiency compared with alternative algorithms like RA, SVR, DT, and GPR, BRANN is the best option because of its capacity to reliably provide highly precise forecasts in real-world situations where accuracy and dependability are critical.

According to the multi-model evaluation shown in Fig. 21, RA and GPR performed well, with low MSE and high R^2^ values. SVR also performs well but significantly less than both RA and GPR. DT has a greater MSE and a lower R^2^, implying lower efficiency. However, BRANN stands out as a top performer, consistently generating precise predictions. The adoption of the BRANN approach seems justified due to its higher performance in correctly estimating pipeline life by obtaining smaller prediction errors based on MSE and R^2^ values. The BRANN's capacity to understand complicated relationships among data, adapt to varied input conditions, and provide accurate predictions makes it ideal for pipeline life prediction. Moreover, the BRANN model's capacity to efficiently manage missing data highlights its dependability and applicability for practical applications in the oil and gas industries. This study could establish an excellent foundation for its approach and give valuable insights into pipeline reliability monitoring by offering an apparent justification for selecting the BRANN framework, among other options.

## Conclusions

This study presents a novel strategy for estimating the life of a dry gas pipeline system while simultaneously detecting the metal loss dimension class detection. The major purpose of this study is to address the problem of predicting the life conditions of dry gas pipelines when missing input variables are present in a dataset for better pipeline system reliability. This study successfully addressed this challenge by introducing a unique BRNN model that manages missing inputs. A few missing input variables were considered for the missing input strategy. A sub-neural network was created for each case to anticipate the missing variables for all four sections of the dry gas pipeline system; the estimated values were then given to the principal BRNNs to estimate the life status of four dry gas pipelines. One of the research's notable findings is the proposed BRNN model's capacity to recognize and detect multiple types of dimension class metal loss inside the pipeline system. In contrast with preexisting models, the proposed BRNN framework proved the capacity to detect the DC metal loss and handle the issue of missing inputs in the dry gas pipeline system. This aspect improves the model's practicality and applicability in real-world situations where corrosion detection and life prediction are critical for determining pipeline integrity. The findings obtained for all four pipeline system sections demonstrated the good performance of the suggested models. The numerical results, i.e. R^2^ and MSE values, were close to 1 and 0 for all four sections of the dry gas pipeline system, demonstrating that the developed models predicted the life condition and dimension class metal loss detection very well. The model's dependability and generalizability were further validated, confirming its usefulness with unseen datasets. This strategic approach of BRANN demonstrates the importance and effectiveness of BR in situations when more than standard methods may be required. In other words, the developed BRANN framework advances a regular BRNN implementation by addressing the dual-objective prediction problem, highlighting the significance and usefulness of BR in instances where standard approaches may be insufficient. Furthermore, this study expands above typical analytic approaches by including multi-model comparison and sensitivity analysis, which justified our developed BRANN's forecast accuracy and gave a deeper understanding of the input factors' significance on pipeline life. This developed framework is most advantageous for avoiding inspection expenses even when missing data. This developed novel approach would benefit oil and gas companies more in terms of pipeline maintenance, leak detection, and improved safety, reliability, and security. In other words, the developed BRNN framework's ability to handle missing inputs and accurately anticipate life conditions and DC metal loss makes it a useful approach for pipeline maintenance and risk assessment. This work enhances awareness and approaches for dealing with missing data in complex systems such as pipeline infrastructure.

## Data Availability

The datasets used and/or analysed during the current study are available from the corresponding author upon reasonable request.

## Abbreviations

ANN: Artificial neural network
MDA: Material degradation analysis
FEA: Finite element analysis
CBM: Condition-based monitoring
ML: Machine learning
AI: Artificial intelligence
PRA: Probabilistic risk assessment
BRNN: Bayesian regularization neural networks
RA: Regression analysis
SVM: Support vector machines
RF: Random forest
DT: Decision trees
RBA: Reliability-based analysis (WA)
SA: Survival analysis
WA: Weibull analysis
BR: Bayesian regularization
ERF: Estimated repair factor
DC: Dimension class
SVR: Support vector regression
POF: Pipeline operator forum
